# Neuromuscular Junction Impairment in Amyotrophic Lateral Sclerosis: Reassessing the Role of Acetylcholinesterase

**DOI:** 10.3389/fnmol.2016.00160

**Published:** 2016-12-27

**Authors:** Maria-Letizia Campanari, María-Salud García-Ayllón, Sorana Ciura, Javier Sáez-Valero, Edor Kabashi

**Affiliations:** ^1^Sorbonne Université, Université Pierre et Marie Curie (UPMC), Université de Paris 06, Unité Mixte 75, Institut National de la Santé et de la Recherche Médicale (INSERM) Unité 1127, Centre National de la Recherche Scientifique (CNRS) Unité Mixte de Recherche 7225 Institut du Cerveau et de la Moelle Épinière (ICM)Paris, France; ^2^Instituto de Neurociencias de Alicante, Universidad Miguel Hernández-CSIC, Sant Joan d’AlacantSpain; ^3^Centro de Investigación Biomédica en Red sobre Enfermedades Neurodegenerativas (CIBERNED)Madrid, Spain; ^4^Unidad de Investigación, Hospital General Universitario de Elche, FISABIOElche, Spain

**Keywords:** amyotrophic lateral sclerosis (ALS), axonopathy, neuromuscular junction (NMJ), acetylcholinesterase (AChE), collagen tail subunit of asymmetric acetylcholinesterase (ColQ)

## Abstract

Amyotrophic Lateral Sclerosis (ALS) is a highly debilitating disease caused by progressive degeneration of motorneurons (MNs). Due to the wide variety of genes and mutations identified in ALS, a highly varied etiology could ultimately converge to produce similar clinical symptoms. A major hypothesis in ALS research is the “distal axonopathy” with pathological changes occurring at the neuromuscular junction (NMJ), at very early stages of the disease, prior to MNs degeneration and onset of clinical symptoms. The NMJ is a highly specialized cholinergic synapse, allowing signaling between muscle and nerve necessary for skeletal muscle function. This nerve-muscle contact is characterized by the clustering of the collagen-tailed form of acetylcholinesterase (ColQ-AChE), together with other components of the extracellular matrix (ECM) and specific key molecules in the NMJ formation. Interestingly, in addition to their cholinergic role AChE is thought to play several “non-classical” roles that do not require catalytic function, most prominent among these is the facilitation of neurite growth, NMJ formation and survival. In all this context, abnormalities of AChE content have been found in plasma of ALS patients, in which AChE changes may reflect the neuromuscular disruption. We review these findings and particularly the evidences of changes of AChE at neuromuscular synapse in the pre-symptomatic stages of ALS.

## Neuromuscular Junction (NMJ) Formation and Stabilization

**Figure 1:**
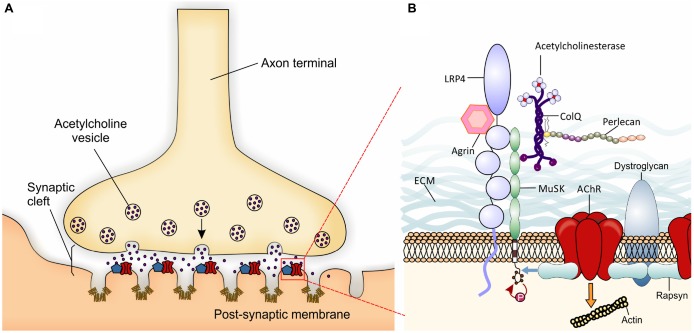
**Schematic presentation of a neuromuscular junction (NMJ) and the molecules involved in its development. (A)** Local specialization in the presynaptic motor nerve terminal where vesicles fuse with the terminal membrane and release Acetylcholine (ACh) neurotransmitter into the synaptic cleft are shown. Postsynaptic organization in the skeletal muscle membrane include several folds with ACh receptors (AChRs; red) at the crest and voltage-gated sodium channels (Nav1.4- brown) in the troughs of the folds. The localization and high concentration of AChR and Nav 1.4 are important for efficient neuromuscular transmission. The entire complex of proteins associated with the AChRs partially represented in B is summarized within the blue pentagon. **(B)** The agrin-Lrp4-MuSK complex is essential for the formation of the NMJ. Neural agrin binds to Lrp4 inducing the activation by phosphorylation of muscle-specific tyrosine kinase receptor (MuSK; red arrow). AChE-ColQ complex is localized to the synaptic basal lamina and is essential for the inactivation of ACh. Also ColQ binds MuSK and Perlecan taking part in the stabilization of the extracellular matrix (ECM). One activated, MuSK binds rapsyn (blue arrow) which in turn links AChRs and dystroglycan. The entire structure is finally attached to the actin cytoskeleton (to simplify the figure, the orange arrow represents the network of proteins and pathways responsible for this interaction) forming the lipid raft at the crest of the muscle membrane folds.

The vertebrate neuromuscular junction (NMJ) is a “tripartite” synapse, composed by the presynaptic motorneuron (MN), the postsynaptic muscle, and the synapse-associated glial cells (terminal Schwann cells, TSC; Castonguay et al., 2001; Jessen and Mirsky, 2005). Thus, the NMJ is a specialized cholinergic synapse that permits the transmission of action potentials from MNs to muscle. Impairment of NMJ function results in muscle weakness or paralysis. In the NMJ, the transmitter acetylcholine (ACh) is released from the MN towards the postsynaptic muscle membrane, folded with crests carrying ACh receptors (AChRs), and troughs with a high density of voltage-gated sodium channels (Flucher and Daniels, 1989; Figure 1A). The muscle fibers are tightly wrapped by a basal lamina containing extracellular matrix (ECM) material (Griffin and Thompson, 2008).

NMJ formation requires interactions between pre- and postsynaptic components during embryonic development. When motor axons reach their final target, muscle fibers already present clusters of AChRs (Yang et al., 2000) which may represent a preferential zone for innervation (Kim and Burden, 2008). Upon contact with muscles, motor axons innervate muscle fibers extensively to generate NMJs (for review Wu et al., 2010). The NMJ specialization is mediated by key molecular mechanisms. Agrin is a glycoprotein synthesized in MNs and released into synaptic clefts. Agrin binds its receptor low-density lipoprotein receptor-related protein 4 (Lrp4) to activate the muscle-specific tyrosine kinase receptor (MuSK). Once phosphorylated, MuSK recruits rapsyn, a cytoplasmic scaffolding protein expressed constitutively in myotubes and tightly bonded to AChRs (Lee et al., 2009). Thus, new AChRs are recruited at the NMJ (Sanes and Lichtman, 2001; Figure 1B). The postsynaptic structure is then maintained by a sub-synaptic apparatus provided by the actin cytoskeleton and the dystrophin-glycoprotein complex (DGC), a transmembrane complex of proteins linking the actin cytoskeleton of the muscle to the basal lamina (Bartoli et al., 2001).

At the NMJ, acetylcholinesterase (AChE) rapidly degrades ACh, thus terminating synaptic transmission (Taylor and Radić, 1994). AChE is presented as a protein complex consisting of three catalytic tetramers covalently linked to a non-catalytic subunit: a three-stranded collagen-like tail, called ColQ (Rotundo, 2003; Massoulié and Millard, 2009). ColQ is composed of a central collagenous domain flanked by non-collagenous N and C-terminus domains (Krejci et al., 1997). ColQ interacts with AChE, through the N-terminus (Bon et al., 1997), to perlecan (a heparan sulfate proteoglycan) through the collagen domain (Peng et al., 1999; Arikawa-Hirasawa et al., 2002) and to MuSK through the C-terminus (Cartaud et al., 2004). In turn, perlecan binds to DGC that, as seen before, links the ECM to the cytoskeleton.

## Acetylcholinesterase (AChE)

Vertebrates possess two types of ChEs: AChE and butylcholinesterase (BChE). AChE is a crucial enzyme for nerve functions, hydrolyzing ACh in the synaptic cleft, while the function of BChE and its role in the regulation of AChE levels is still under discussion.

AChE exists as three distinct variants coming from alternative exon splicing, each of them with a different C-terminal sequence which determines the possibility to form different oligomeric species (Taylor and Radić, 1994; Massoulié, 2002; Meshorer et al., 2004). The main AChE mRNA transcript in brain and muscle tissues is the AChE-T (tail) variant which subunits constitute the AChE-ColQ complexes. Alternative AChE-H (hydrophobic) and AChE-R (readthrough) variants are less represented. Based on their quaternary structure and on hydrodynamic properties, AChE-T appears under several globular forms: amphiphilic G1, amphiphilic G2, non-amphiphilic G4 and the membrane anchored amphiphilic tetramers bound through the transmembrane subunit Proline Rich Membrane Anchor (PRiMA). The main form at NMJs is asymmetrical, consisting of 1 or 3 tetramers of AChE (A4, A8 or A12) attached to ColQ (for review, see Massoulié et al., 2005). In addition, three more variants exist coming from the 5′-end splicing (Meshorer et al., 2004), called “N-extended” species: N-AChE-T, N-AChE-H and N-AChE-R (Muñoz-Delgado et al., 2010). It results in a complex scenario where multiple forms of the same protein may be regulated by different mechanisms and display different biological roles.

## AChE-ColQ Complex at the NMJ

ColQ deficiency brings to a drastically decreased AChE localization at NMJs (Feng et al., 1999), causing congenital myasthenic syndrome with AChE deficiency (Donger et al., 1998; Ohno et al., 1998, 2013; Schreiner et al., 2007). However, despite its cholinergic role, AChE-ColQ exerts multifunctional roles thanks to the ability of ColQ to binds several partners. Interestingly, AChR clusters are smaller and more densely packed in ColQ-deficient mouse in both muscle cells and *in vivo* NMJs (Sigoillot et al., 2010). In addition, recent *in vitro* studies in absence of ColQ revealed the down regulation of several ECM mRNAs (Sigoillot et al., 2016). Laminins, collagens, proteoglycans and other glycoproteins, but also metalloproteases, and other modulators are decreased in ColQ^−/−^ myotubes. Thus, ColQ may modify the postsynaptic differentiation through the regulation of major ECM components.

Also AChE regulates the fate of AChR: in AChE mutant mice the absence of AChE causes a decrease in AChRs density (Adler et al., 2004). Interestingly, BChE can associate with ColQ (Krejci et al., 1997; Feng et al., 1999), but its depletion does not give any phenotype at the NMJ (Li et al., 2006), confirming the specificity of AChE action. Although the catalytic action of AChE in the nervous system has been characterized many years ago, its role in development remains enigmatic. Many evidences, led to the hypothesis that AChE may play *non-classical* roles, which can be relevant during neural development. In this regard, it has been shown that AChE expression occurs largely before the onset of synaptogenesis, and in the absence of ACh (Layer and Kaulich, 1991; Small et al., 1992, 1995). Moreover, AChE shares sequence homologies with several cell-adhesion proteins, called cholinesterase-like proteins, catalytically inactive but implicated in protein–protein interactions (de la Escalera et al., 1990; Krejci et al., 1991; Grifman et al., 1998; Grisaru et al., 1999). The existence of these proteins provided a convincing reason to assume that AChE itself may be engaged in protein interactions contributing to the formation of cellular junctions by binding other extracellular ligands.

Zebrafish has provided an excellent system to investigate the roles of AChE *in vivo* due to the absence of BChE gene (Bertrand et al., 2001), while AChE gene is widely expressed throughout development (Hanneman and Westerfield, 1989). A missense mutation in the zebrafish AChE gene, *achesb55*, was able to abolish ACh hydrolysis. The mutant embryos showed a progressive motility defect and severe reductions in the formation of muscle AChR clusters (Behra et al., 2002). They also had defects in muscle fiber development, with decrease in primary sensory neuron survival and dendritic growth. These defects collectively supported non-classical roles of AChE. Another mutation on AChE gene in zebrafish, the *zim (ach)*, gave rise to a protein, lacking both the catalytic site and the C-terminal neuritogenic domain (Downes and Granato, 2004). This mutation did not cause any motor projections deficit, demonstrating that AChE activity was not required for motor axon growth. However, both mutants did display a decrease in AChR clusters at NMJ. Further experiments on mutant mice presenting deletions of exons 5 and 6 in the AChE gene in the muscles, (mutation which transforms anchored AChE into a soluble enzyme (Camp et al., 2005), with consequent absence of AChE from the NMJ basal lamina (Girard et al., 2006)), were presenting AChR clusters markedly fragmented with a tetanic fade of muscles contraction (Girard et al., 2006). Taken together, these results demonstrated that AChE is dispensable for regulating the stability of neuromuscular synapses. However, the molecular mechanisms behind this regulation remain unknown.

## Early Sign for NMJ Destruction in Amyotrophic Lateral Sclerosis (ALS)

Amyotrophic Lateral Sclerosis (ALS) is caused by progressive degeneration of upper and lower MNs with rapid clinical course. Denervated muscles weaken, atrophy and death usually occurs due to respiratory failure in patients within 2–5 years. Despite our knowledge above the genetic causes for this disease, a major question is still opened: Where does MN dysfunction begin? Both dying-forward and dying-back hypothesis have been considered (Kiernan et al., 2011). While the first one proposes an anterograde degeneration of MNs via glutamate excitotoxicity from the cortex, the second raises the possibility that ALS starts distally at the nerve terminal or at the NMJ and progress towards the cell body. In this complicated scenario, the best is to consider back and forward-dying processes as two independent mechanisms that happen simultaneously.

Due to the difficulties in studying human cases, because of the impossibility of obtaining pathological presymptomatic samples, transgenic mice expressing human mutated genes have provided an opportunity to investigate the early stages of the disease. In particular, the human SOD1^G93A^ transgenic mouse is the most commonly used model of ALS (Dupuis and Loeffler, 2009). This animal loses MNs, develops progressive paralysis and dies at 4–5 months of age (Gruzman et al., 2007; Turner and Talbot, 2008). Thus, using this model Fischer et al. (2003) observed denervation at the NMJ by day 47, followed by loss of motor axons between days 47 and 80, and loss of MNs cell bodies after day 80. Interestingly, MNs in hSOD1^G93A^ mice showed diminished retrograde uptake and transport before the manifestation of clinical ALS symptoms (Parkhouse et al., 2008; Bilsland et al., 2010). These results, plus other relevant data discussed by Dadon-Nachum et al. (2011), implied that hSOD1^G93A^ dysfunction is actually a “dying-back” phenomena where distal components are affected early before neuronal degeneration, and symptoms onset.

Also, several results suggested that synapse-specific mechanisms are partly responsible for selective synaptic loss before MNs degeneration. MNs are generally divided into two classes: fast-fatigable (FF) and slow (S) (Heckman and Enoka, 2012). A third kind of MNs exists, the fast-fatigue-resistant (FFR), considered an intermediate phenotype between FF and S MNs, but little is known about its physiology (Stifani, 2014). In hSOD1^G93A^ mouse the FF MNs synapses degenerates while S-MNs synapses remain preserved (Pun et al., 2006). Also the whole muscle and motor unit isometric contractile forces are reduced, 50 days before the onset of clinical symptoms (Hegedus et al., 2007).

Interestingly, the high constitutive expression of hSOD1^G93A^ at the MNs alone is not sufficient to develop early onset of ALS in mouse (Pramatarova et al., 2001; Lino et al., 2002). Notably, the transgenic overexpression of hSOD1^G93A^ at the skeletal muscle, develops progressive muscle atrophy with mitochondrial dysfunction (Dobrowolny et al., 2008); suggesting the role of muscles in disease initiation. This potential role is highlighted by the therapeutic benefit of muscle-targeted treatments. Indeed, the muscle expression of neurotrophic factors delays disease onset, improves locomotor performance, and increases lifespan (Mohajeri et al., 1999; Acsadi et al., 2002; Azzouz et al., 2004; Dobrowolny et al., 2005; Li et al., 2007). Very recently, morphological and pathological changes at hSOD1^G93A^ mouse NMJs architecture have been described as pre-symptomatic hallmarks of the disease (Clark et al., 2016). These observations point to an active role of the muscle fiber and identify the NMJ as a major player in the initiation and progression of ALS.

Further evidences of early dysfunction at the NMJ come from the FUS (*FU*sed in Sarcoma) mutant mice (Sharma et al., 2016). In particular, in the hFUS^P525L^ mouse lines, where the human FUS^P525L^ (found in patients with an aggressive and juvenile-onset form of ALS) mutation is conditionally expressed in MNs, the progressive degeneration is preceded by early (day p20) and selective (FF MNs) motor axons retraction. Also, the NMJs are precociously changed with a drastic reduction of synaptic vesicles and pre- and postsynaptic mitochondria, which appeared dilated and vacuolated as described in mutant hSOD1^G93A^ and hTDP43^A315T^ transgenic models of ALS (Magrané et al., 2013).

Very recently, an additional mouse model for ALS has been described carrying the human mutated C9orf72 with the hexanucleotide repeat-expansion at the first intron/promoter (gain-of-function model; O’Rourke et al., 2015). These mice display early nuclear RNA foci and DPR proteins accumulation without showing any sign of neurodegeneration. Also zebrafish partial loss-of-function C9orf72 model have been described leading to motor deficits (Ciura et al., 2013). Probably, C9orf72 models recapitulate the presymptomatic phase of disease which needs of additional genetic factors to manifest the neurodegeneration (Sellier et al., 2016). Probably, it will be an extremely useful model for studying early stages of disease pathogenesis.

## Cholinergic Dysfunction in ALS

The cholinergic system deficit emerged as a common pathological hallmark in various neurodegenerative diseases, such as Alzheimer’s disease (AD; Ulrich et al., 1990; Inestrosa et al., 1996; Sáez-Valero et al., 1999), Parkinson’s disease (PD; Bosboom et al., 2003), dementia with Lewy bodies (Förstl et al., 2008), subcortical vascular dementia (Amenta et al., 2002) and Huntington’s disease (Smith et al., 2006). Consequently, AChE attracted considerable attention as a potential therapeutic target (Fernandez et al., 2000; Erkinjuntti et al., 2004; Rampello et al., 2004; Maidment et al., 2005). Indeed, AChE inhibitors partially improve cognitive and functional symptoms, by increasing the synaptic ACh availability. However, little is known about its implication in ALS.

Loss of cholinergic synapses was reported in sporadic ALS patients by studying the expression of vesicular ACh transporter (VAChT), involved in the packaging of ACh inside the synaptic vesicles before release. This protein is localized at the synaptic terminal and considered a marker for cholinergic synapses. Immunohistochemical exams on the spinal MNs revealed a drastic depletion of VAChT immunoreactivity respect to synaptophysin, a marker for MNs synapses integrity. This discrepancy suggested a loss of cholinergic inputs as an early event of ALS neurodegeneration (Nagao et al., 1998). However, the choline acetyltransferase (ChAT) enzyme, responsible for the biosynthesis of ACh, is the most specific indicator for monitoring the cholinergic functional state. Microassay analysis of ChAT activity of single spinal MNs from ALS patients showed lower ChAT activity than in control neurons at an early stage of the disease (Kato, 1989). The loss of activity can be explained by the low ChAT protein contents in ALS preserved MNs (Oda et al., 1995). Recently, to unravel how and when cholinergic function is compromised, the spatiotemporal expression of ChAT from early presymptomatic stages of the hSOD1^G93A^ mouse model, has been analyzed by confocal immunohistochemistry. ChAT content was clearly reduced in MNs soma and cholinergic synaptic terminals very early, before MNs loss and NMJs detachment (Casas et al., 2013). Thus, ChAT reduction may contribute to distal degeneration.

Interestingly, muscle biopsies of ALS patients revealed a reduction in the AChE level (Rasool et al., 1983). Subsequently, analysis of plasma AChE in ALS patients revealed a huge increase in the circulating enzyme (Festoff and Fernandez, 1981). Such a release may reflect a disruption of extracellularly bound AChE at the NMJ. However, the exact source of AChE increase in ALS plasma remains uncertain. In 1983, this hypothesis was confuted by a study were ALS patients plasma samples were compared with neuromuscular disease control groups (which included 2 patients with denervating illnesses). However, no increase of AChE activity was noticed in the second group, suggesting that the increase in AChE activity in ALS plasma was unlikely due to a release of bound enzymes from the NMJ (Rasool et al., 1983). The difficulty to determine human plasma AChE levels without interference by BChE may also explain discrepancies (García-Ayllón et al., 2010, 2012). More information comes from studies in which nerve-muscle integrity was altered, such as denervation or dystrophy, where it has been proved a similar reduction of AChE at the animal NMJ with a subsequent increase in the plasma (Wilson et al., 1973; Fernandez and Inestrosa, 1976). Further data evidence that matrix metalloproteinases (MMP) activity increased in central nervous system as well as in muscles (Schoser and Blottner, 1999) and plasma of ALS patients (Demestre et al., 2005). MMP are a family of Zn^2+^ endopeptidases that are characterized by their ability to digest components of the ECM, such as collagen, proteoglycan, and laminin (Vincenti and Brinckerhoff, 2007) in response to specific changes in neuronal activity or diseases (reviewed by Reinhard et al., 2015). Also, a study on SOD1^G93A^ mice model demonstrated that early treatment with an MMP inhibitor prolongs survival, suggesting a role for MMPs in disease progression, since treatment in the symptom-onset group did not significantly prolonged survival (Lorenzl et al., 2006). We can speculate that such an increase of proteases and collagenases may be related with the *in vitro* release of AChE found with denervation and denervating illnesses and could be partly the cause of the continuous and progressive interruption of neuromuscular integrity and interrelationships intimately involved in the pathogenesis of ALS.

However, the muscle is not the unique source of AChE in the plasma: MNs have been shown to produce and release AChE (Juliana et al., 1977; Rodríguez-Ithurralde et al., 1997). To complicate the scenario there is the recent discovery of BChE anchored by PRiMA on the surface of TSCs at mouse NMJs (Petrov et al., 2014). In conclusion, the cellular origin of the AChE released in the plasma in ALS and the consequences of its absence at the NMJ is still unclear, since many other functions, not related to the ACh hydrolysis, have been described (Soreq and Seidman, 2001).

## Conclusion

Although the majority of ALS cases are sporadic ALS (sALS) with an unknown etiology, in about 10% of the cases there is a Mendelian inheritance (fALS) where more than 20 genes seems to be implicated (Lattante et al., 2015). Beyond these genes, a huge interested has been put on TARDBP gene, because its protein, TDP-43, is involved in multiple steps of RNA metabolism, including transcription, splicing, or transport of several mRNAs (Lagier-Tourenne et al., 2010; Lattante et al., 2013). Interestingly, ChAT mRNA is a target of TDP-43 (Buratti et al., 2010) and TDP-43 levels and localization in all the spinal MNs are severely affected early in the presymptomatic stage in hSOD1^G93A^ mice, and parallels the development of cholinergic dysfunctions (Casas et al., 2013). In this regard, we can speculate a possible implication for TDP-43 in the direct regulation and dysregulation of AChE or ColQ/PRiMA in ALS. In the same way, would be of great interest to better explore the cholinergic deficit in others, less known, genetical ALS models to give further clues onto the etiopathogenesis of the diseases and to translate data in validation of early biomarkers.

## Author Contributions

Conception and drafting by M-LC. The work was coordinated, revisited and approved by EK, M-SG-A, SC and JS-V.

## Funding

This study was funded by the CIBERNED, Instituto de Salud Carlos III (ISCIII; Cooperative project PI2015/01-4, to JS-V), Spain; Atip/Avenir from Inserm, Career Integration Grant (Marie Curie Actions), Robert Packard Foundation, E-rare ERA-NET program, AFM, ARSLA, France-Alzheimer association and the program “Investissements d’avenir” ANR-10-IAIHU-06 (EK).

## Conflict of Interest Statement

The authors declare that the research was conducted in the absence of any commercial or financial relationships that could be construed as a potential conflict of interest.
